# Development of a novel‐type transgenic cotton plant for control of cotton bollworm

**DOI:** 10.1111/pbi.12534

**Published:** 2016-02-03

**Authors:** Zhen Yue, Xiaoguang Liu, Zijing Zhou, Guangming Hou, Jinping Hua, Zhangwu Zhao

**Affiliations:** ^1^ College of Agriculture and Biotechnology China Agricultural University Beijing China

**Keywords:** transgenic plant, neuropeptide F, cotton budworm, *Helicoverpa armigera*

## Abstract

The transgenic Bt cotton plant has been widely planted throughout the world for the control of cotton budworm *Helicoverpa armigera* (Hubner). However, a shift towards insect tolerance of Bt cotton is now apparent. In this study, the gene encoding neuropeptide F (NPF) was cloned from cotton budworm *H. armigera*, an important agricultural pest. The *npf* gene produces two splicing mRNA variants—*npf1* and *npf2* (with a 120‐bp segment inserted into the *npf1* sequence). These are predicted to form the mature NPF1 and NPF2 peptides, and they were found to regulate feeding behaviour. Knock down of larval *npf* with dsNPF *in vitro* resulted in decreases of food consumption and body weight, and dsNPF also caused a decrease of glycogen and an increase of trehalose. Moreover, we produced transgenic tobacco plants transiently expressing dsNPF and transgenic cotton plants with stably expressed dsNPF. Results showed that *H. armigera* larvae fed on these transgenic plants or leaves had lower food consumption, body size and body weight compared to controls. These results indicate that NPF is important in the control of feeding of *H. armigera* and valuable for production of potential transgenic cotton.

## Introduction

The cotton bollworm (*Helicoverpa armigera*) is an important agricultural pest and is responsible for great losses of cotton production. More than one and a half billion US dollars was lost in China in 1992 due to its outbreak. The transgenic *Bacillus thuringiensis* (Bt) cotton expressing the Cry1Ac toxin protein is being used for insect control. The report from the International Service for the Acquisition of Agricultural‐biotechnology applications showed that the planting area of the transgenic Bt cotton continuously increased in the last 15 years and attained 160 million hm^2^ in 2011, about half of the total cotton planting area in the world (James, 2012). A shift towards insect tolerance of Bt cotton is now apparent, indicating that potential resistance from the target pest—the cotton bollworm—has become a major threat for sustainable planting of Bt cotton (Kong‐Ming, 2007). For example, a report from the Chinese Agriculture Department showed that the yield of Bt cotton decreased around 10% in China in 2009 due to the development of resistance by cotton bollworm. Recent monitoring records indicated that the increased tolerance of cotton bollworm was apparent year by year in the intensive planting area of Bt cotton. So, it is imperative to develop new types of transgenic cotton (Zhang *et al*., 2010).

Insect brain neuropeptides are important regulators of physiology and behaviour. Neuropeptide F (NPF), a family member of neuropeptide Y (NPY) of vertebrates (Huang *et al*., 2011; Nuss *et al*., 2010; Roller *et al*., 2008) because of their similar function and similar signalling path via G protein‐coupled receptors (Garczynski *et al*., 2005), has pleiotropic functions. However, because of fast evolution, their peptide sequences and structures differ greatly among animal species.

The NPFs have not been widely reported in insects. NPF was first isolated from *Helicoverpa zea* (Huang *et al*., 1998), followed by *Drosophila melanogaster* (Brown *et al*., 1999), *Schistocerca grearia* (De Loof *et al*., 2001), *Locusta migratoria* (Clynen *et al*., 2006), *Aedes aegypti* (Stanek *et al*., 2002), *Anopheles gambiae* (Garczynski *et al*., 2005), *Bombyx mori* (Roller *et al*., 2008), *Reticulitermes flavipes* (Nuss *et al*., 2010) and *Helicoverpa assulta* (Liu *et al*., 2013). Extensive studies on NPF have focused on the fruit fly (*D. melanogaster*) as a model. In brief, NPF exerts diverse regulatory roles in feeding (Lingo *et al*., 2007; Wu *et al*., 2005), ethanol sensitivity (Wen *et al*., 2005), learning and memory (Krashes *et al*., 2009), aggression (Dierick and Greenspan, 2007), locomotor circadian rhythms and sleep (He *et al*., 2013a,b). However, there are no reports on NPF about the regulation of feeding behaviour in important agricultural and economical pests. Does NPF exist in *H. armigera*? Does it regulate feeding behaviour? Might it be used for controlling pests in the field through transgenic biotechnology? To answer these questions, we identified and cloned the NPF gene from the cotton bollworm (*H. armigera*), analysed its feeding function, and found a new way to efficiently control this pest using transgenic crops expressing dsNPF RNAi.

## Results

### Identification and cloning of *Harmnpfs* and formation of the mature peptides

**Figure 1:**
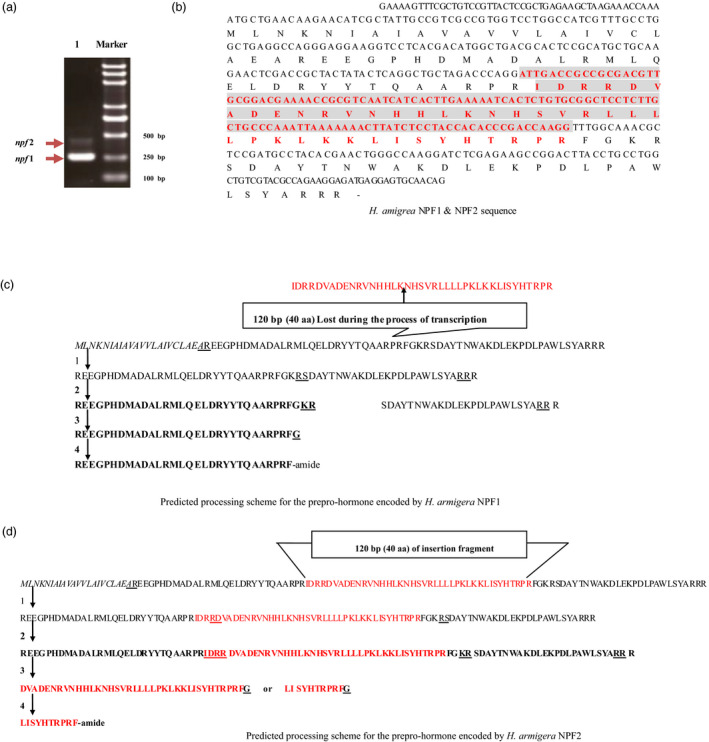
Structure of *Harmnpf1/Harmnpf*2. (a) The *Harmnpf1* and *Harmnpf2* mRNAs by PCR, in which Marker indicates standard molecular weight and 1 indicates PCR bands representing *Harmnpf1* and *Harmnpf2*. (b) Nucleotide sequences for the ORF encoding *Harmnpf1* and *Harmnpf2*. *Harmnpf2* includes a 120‐bp sequence (red colour fragment) not found in *Harmnpf1*. (c, d) Separately indicates the predicted processing scheme for the preprohormone that is encoded by *Harmnpf1* and *Harmnpf2*.

The constructed non‐normalized cDNA libraries from the brain tissues of *H. armigera* were isolated and collected with CHROMA SPIN‐400 columns (Clontech, Laboratories, Inc., Mountain View, CA). The insert fragments of recombinant plasmids from bacterial colonies ranging from 400 to 1200 bp were separated on a 1% agarose gel (Biowest, Gene Company LTD, Chai Wan, Hong Kong), and the percentage of recombinants selected from 150 independent clones (133 positive clones) was 95%. Subsequently, the two *npf*s in *H. armigera* were identified, isolated and cloned (Figure 1a), and the sequences were deposited in the GenBank (*Harmnpf*1 accession number HQ613404; *Harmnpf*2 accession number HQ416718). *Harmnpf*1 and *Harmnpf*2 contain an ORF of 246 and 366 bp, respectively (Figure 1b), in which a 120‐bp insertion segment between 135th and 136th nucleotides of *Harmnpf*1 forms *Harmnpf*2. The predicted mature neuropeptides *HarmNPF*1 and *HarmNPF*2 are composed of 30 and 10 amino acids, respectively, formed by a series of proteolytic processes and post‐translational modifications such as TrimKR and Amidation (Figure 1c,d) through sequential action of three enzymes (prohormone convertases for TrimKR; peptidyl‐hydroxylating monooxygenase and peptidyl‐hydroxyglycine a‐amidating lyase for amidation; McVeigh *et al*., 2005).

### Temporal and spatial expression of *Harmnpf*

**Figure 2:**
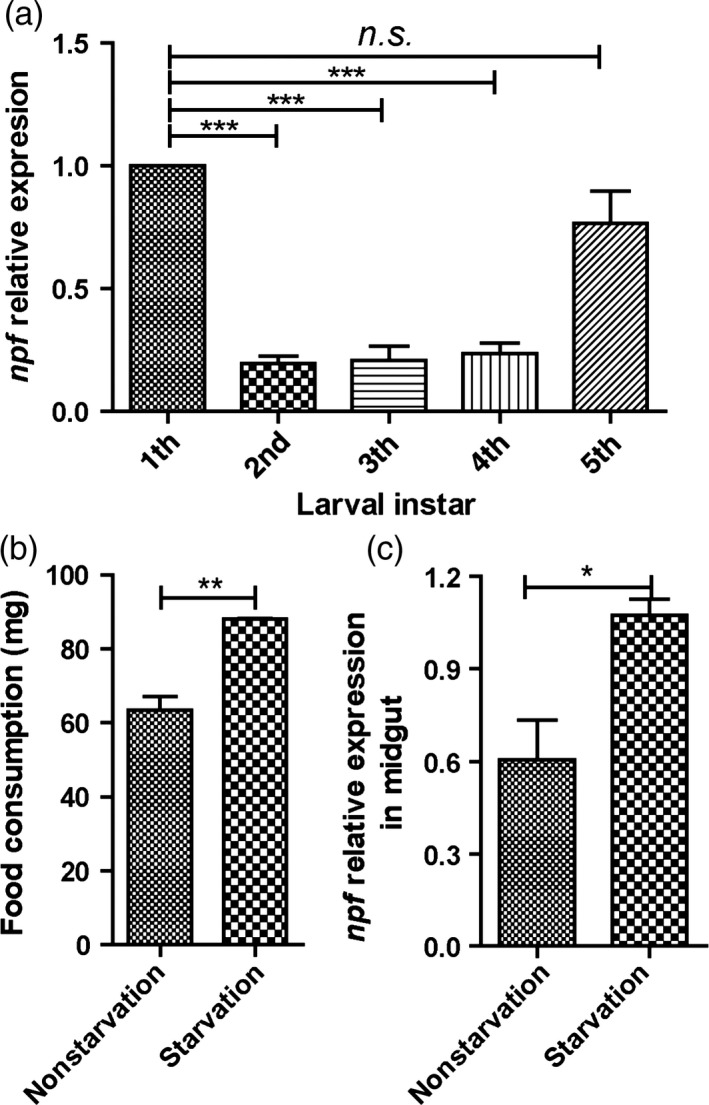
Relative expressions of *Harmnpf* at larval instar and effects of starvation treatment. (a) The relative expression level of *Harmnpf* at different larval instar stages (*n* = 30). n.s., no significant difference. (b) One‐hour food consumption after 24‐h starvation or no starvation. (c) The NPF relative expression levels after 24‐h starvation treatment or no starvation (*n* = 10). All data are expressed as the mean ± SD of three replicates. Differences at ”*”*P* < 0.05, ”**”*P* < 0.01 and ”***”*P* < 0.001.

Spatial expressions of *Harmnpf1* and *Harmnpf2* mRNA in selected tissues were investigated by RT‐PCR. Results showed that the transcript levels of *Harmnpf1* were detected in almost all tissues, and transcript levels of *Harmnpf2* were mainly expressed in midgut (MG), suboesophageal ganglia (SG) and thoracic ganglia (T) (Figure S1). Moreover, the expression levels of *Harmnpf* (*Harmnpf2* was selected for the qPCR throughout the manuscript) at different larval stages were also measured, and results showed that expression levels of *Harmnpf* during the first instar (just after emerging as larvae from egg shells) and last instar (the period for gluttony) were much higher than the other stages (Figure 2a). When larvae were starved for 24 h at beginning of the last instar, their feeding was stimulated and promoted with significant increases of the *Harmnpf* levels (Figure 2b,c). These results together suggest that *npf* is involved in modulating feeding behaviour.

### Larval feeding inhibition by injection of dsNPF

**Figure 3:**
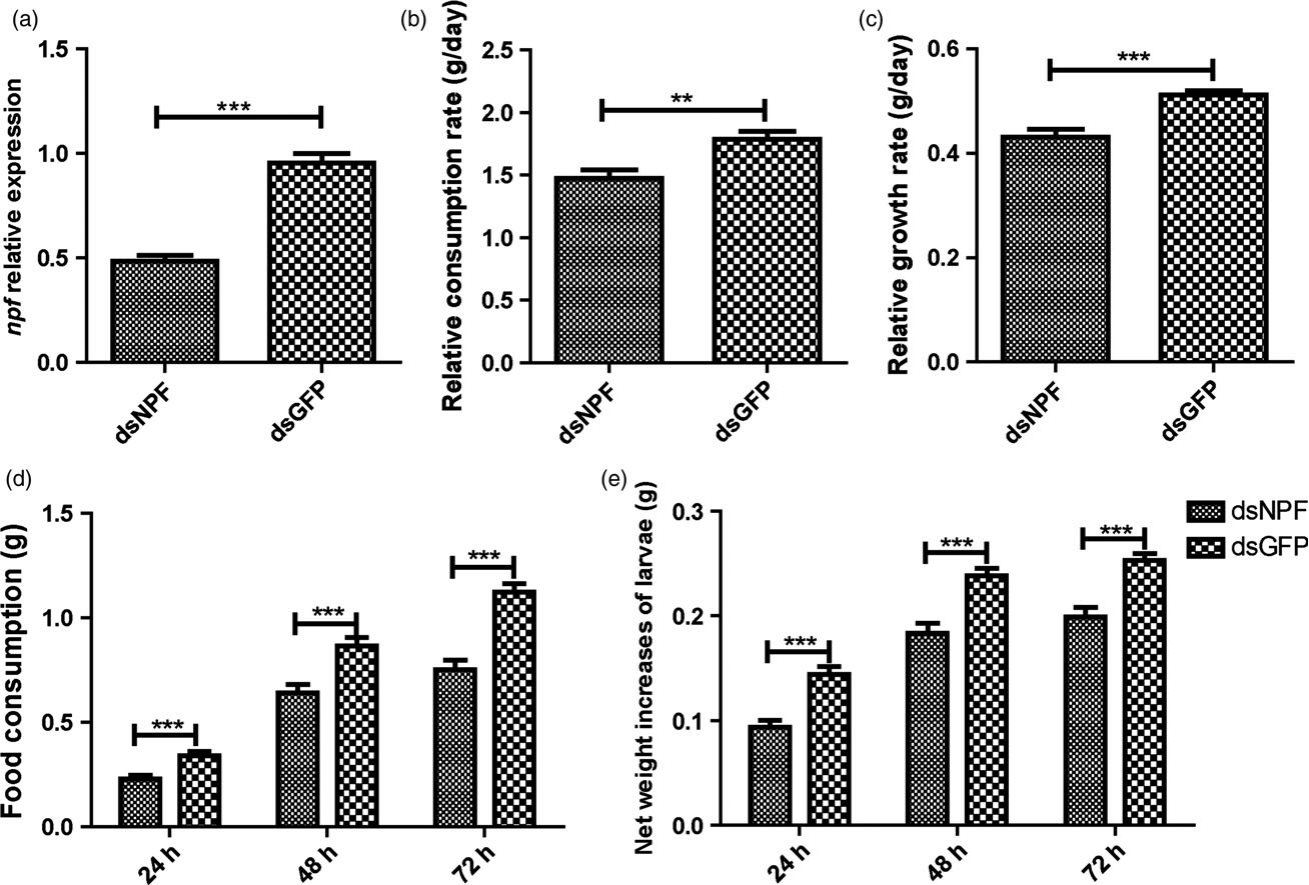
Impact of feeding on larvae treated with dsNPF. The dsNPF or dsGFP was injected to fifth‐instar larvae at the first day. (a) The expressions of NPF transcripts after injection. Green Fluorescent Protein (GFP) is a negative a control. (b) Larval relative consumption rates. (c) Larval relative growth rates. (d) Larval food consumption. (e) Larval net weight increase. All data are expressed as the mean ± SD of three replicates. Differences at ”*”*P* < 0.05, ”**”*P* < 0.01 and ”***”*P* < 0.001.

The dsNPF was designed with consensus primers of *npf1* and *npf2*. Injection of dsNPF RNAi to the fifth instars significantly reduced larval *npf* mRNA levels (Figure 3a). Also, the relative consumption rate of food (Figure 3b; Table 1) and the relative growth rate (Figure 3c; Table 1) in the dsNPF‐treated larvae were significantly lower than in control larvae (*P* < 0.001 and *P* < 0.001, respectively, compared to dsGFP RNAi controls). The amounts of food consumption in dsNPF RNAi‐treated larvae from 24 to 72 h after injection were significantly lower than in the controls, demonstrating a 33% (*P* < 0.001), 26% (*P* < 0.001) and 33% (*P* < 0.001) decrease at 24, 48 and 72 h, respectively (Figure 3d; Table 2). And the larval net weight was also significantly reduced by 35% (*P* < 0.001), 22.89% (*P* < 0.001) and 21.45% (*P* < 0.001) at 24, 48 and 72 h, respectively (Figure 3e; Table 1). These results together indicate that NPF significantly regulates feeding.

**Table 1:** Effect of feeding dsNFP to larvae

	Injected dsNPF	Injected dsGFP	*P* value	Decrease %
NPF relative expression level (%)	0.48 ± 0.03	0.95 ± 0.07	0.013	49.25
	*n* = 3	*n* = 3		
Relative consumption rate (g/day)	1.47 ± 0.07	1.79 ± 0.06	0.001	17.61
	*n* = 38	*n* = 37		
Relative growth rate (g/day)	0.43 ± 0.02	0.51 ± 0.01	<0.0001	15.77
	*n* = 38	*n* = 36		
Food consumption (g)				
24 h	0.23 ± 0.02	0.34 ± 0.02	<0.0001	32.58
	*n* = 39	*n* = 35		
48 h	0.64 ± 0.04	0.87 ± 0.04	<0.0001	25.96
	*n* = 39	*n* = 35		
72 h	0.75 ± 0.05	1.12 ± 0.04	<0.0001	32.94
	*n* = 39	*n* = 35		
Net weight increases of larvae (g)				
24 h	0.10 ± 0.01	0.14 ± 0.01	<0.0001	35.00
	*n* = 36	*n* = 34		
48 h	0.18 ± 0.01	0.24 ± 0.01	<0.0001	22.89
	*n* = 36	*n* = 34		
72 h	0.20 ± 0.01	0.25 ± 0.01	<0.0001	21.45
	*n* = 36	*n* = 34		

**Table 2:** Larval feeding inhibition on dsNPF transgenic tobaccos

Area of leaf eaten (cm^2^)	dsGFP	dsNPF	*P* value	Decrease %
	*n* = 9	*n* = 9		
24 h	21.50 ± 1.92	16.36 ± 0.96	0.044	23.80
48 h	37.13 ± 2.56	18.41 ± 1.94	<0.0001	50.41
72 h	23.11 ± 1.87	16.36 ± 1.04	0.013	29.23

### Larval feeding inhibition on dsNPF transgenic tobaccos

**Figure 4:**
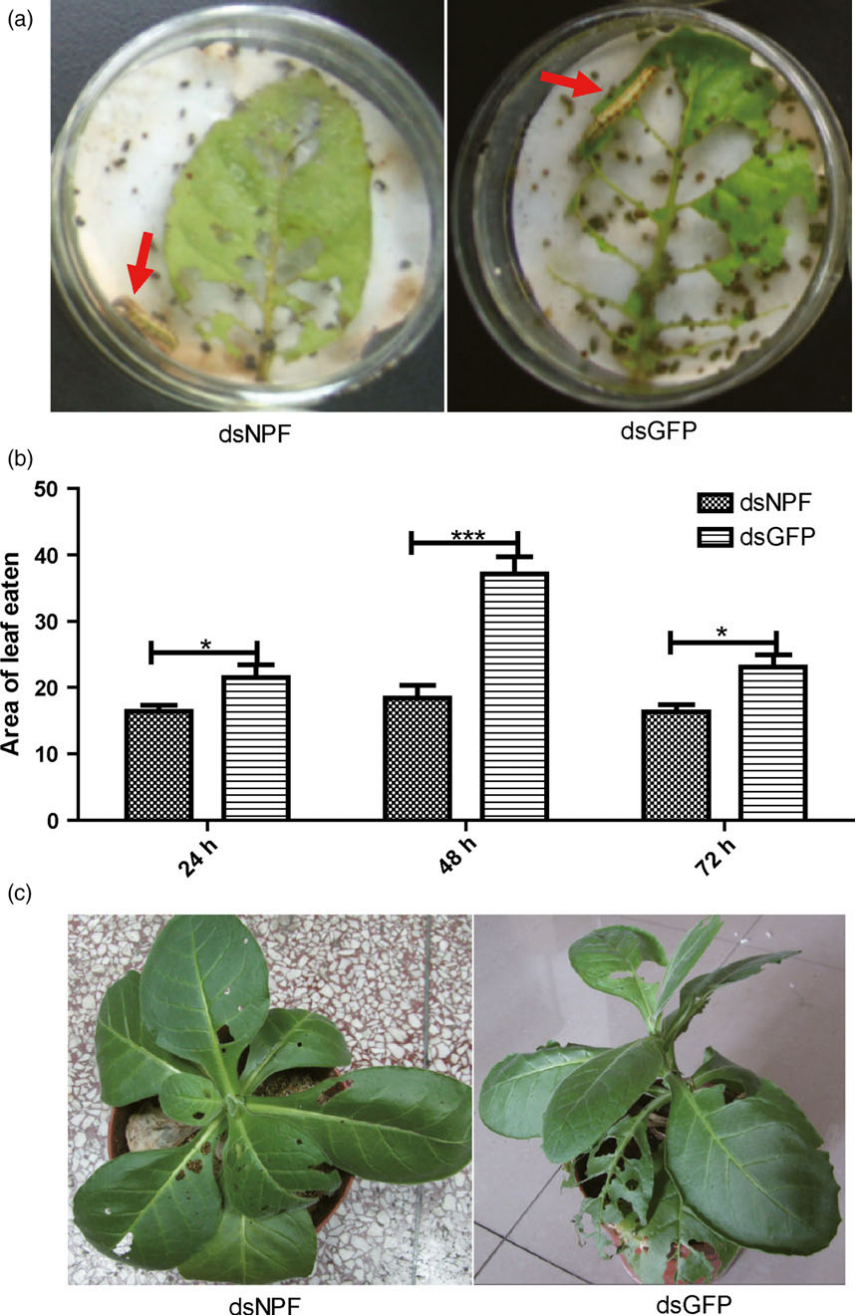
Larvae feeding inhibition by transgenic tobacco expressing dsNPF. (a) The larvae feeding on transgenic tobacco leaves and controls. (b) The leaf areas produced by larvae feeding on transgenic and control leaves separately at 24, 48 and 72 h (*n* = 9). (c) Damages of leaves by feeding whole tobacco plants at 72 h (*n* = 9). All data were expressed as the mean ± SD of three replicates. Differences at “*”*P* < 0.05, “**”*P *< 0.01 and ”***”*P* < 0.001.

The dsNPF was concatenated into the pGreen‐HY104 vector (Figure S2a), identified by PCR and transfected into tobacco protoplasts by osmosis of tobacco leaf for transient expression of dsNPF. After 1 week, the protoplasts were separately identified by PCR for transfected dsNPF (Figure S2b), and the successful transgenic tobaccos, which were identified and determined by northern blot analysis (Figure S3a), were assayed for larval feeding after 2 weeks. When each larva was separately added to a transgenic tobacco leaf expressing dsNPF in a *Petri* dish, results showed that larval feeding was significantly reduced compared to dsGFP controls (Figure 4a). The areas of leaf eaten by larvae at 24 h (*P* < 0.05), 48 h (*P* < 0.001) and 72 h (*P* < 0.01) were significantly less than those of controls (Figure 4b; Table 2). When each larva was directly applied to a transgenic tobacco plant, the results were similar to those above; the larvae fed more on transgenic dsGFP controls than on transgenic tobacco plants (Figure 4c). These results together indicate that NPF in *H. armigera* regulates larval feeding behaviour.

### Larval feeding inhibition on transgenic cotton plants expressing dsNPF

**Figure 5:**
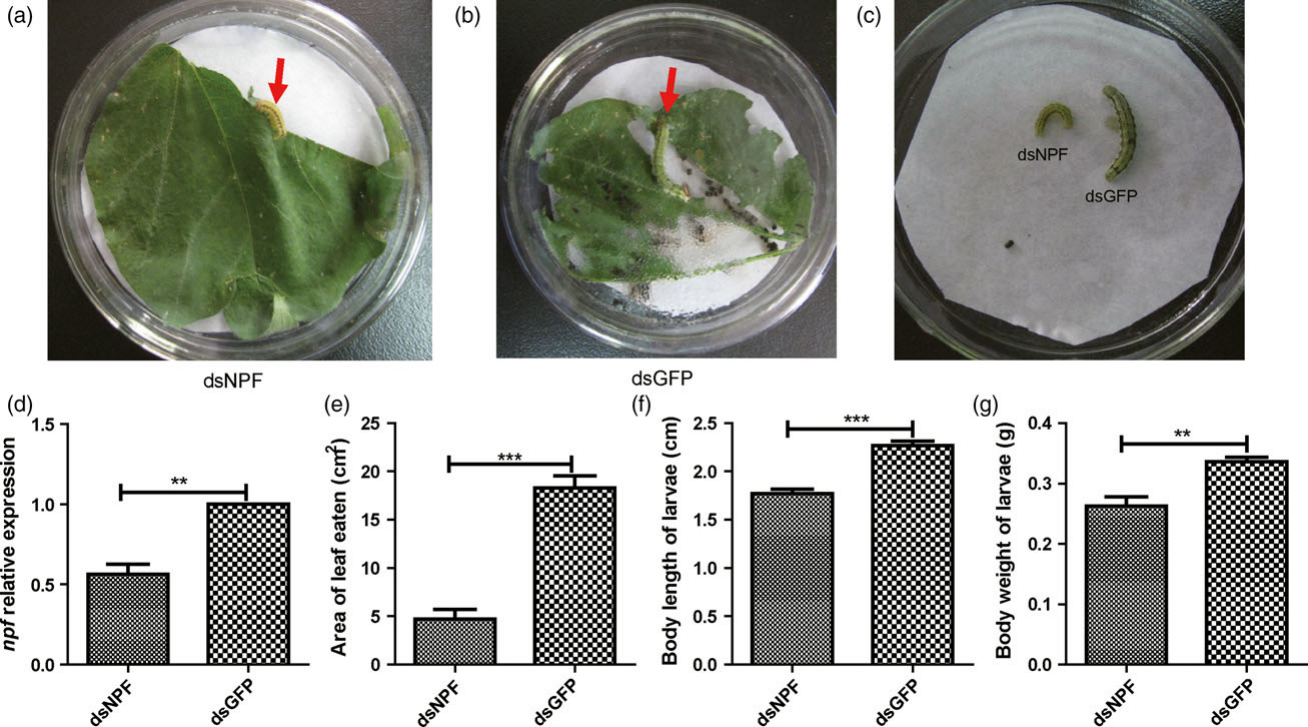
Larvae feeding inhibition by transgenic cotton expressing dsNPF. Ten larvae were released on top of mature leaves to feed on the transgenic plant for 3 days (*n* = 9). (a) Larvae feeding on the dsNPF transgenic cotton leaf. (b) Larvae feeding on the dsGFP (Green Fluorescent Protein) transgenic cotton leaf (a negative control). (c) Comparison of larvae growth with feeding on transgenic cottons. (d) the NPF expression levels of larvae fed on transgenic cottons. (e) A leaf area comparison of larval feeding on dsNPF and dsGFP RNAi transgenic cottons. (f) A comparison of larvae body length after feeding on the transgenic cottons. (g) A comparison of larvae body weight after feeding on the transgenic cottons. Differences at “*”*P* < 0.05, “**”*P* < 0.01 and “***”*P* < 0.001.

The dsNPF cloned in the DH5a plasmid was transformed into the cotton 2047B through hypodermic injection for stable expression of dsNPF. Their seeds were grown until the two‐leaf stage used for the identification of the positive cottons by PCR. The identified NPF‐positive and the dsGFP transgenic cotton as control were further grown. Larvae were added to the mature transgenic cotton leaves, further identified by northern and Southern blots (Figure S3a,b), and incubated in *Petri* dishes (one larva/one leaf/petri dish) for feeding assays. Results showed that the leaf area of the dsNPF transgenic cotton eaten by larva was much less (Figure 5a,e; Table 3) than that of the dsGFP transgenic cotton as a control (*P* < 0.001; Figure 5b,e; Table 3). The larval body size and weight after feeding transgenic leaves were also significantly smaller than for the controls (*P* < 0.01; Figure 5c,f,g; Table 3). Moreover, the *npf* level in the larvae after feeding dsNPF cotton leaves was significantly lower than that in control larvae (*P* < 0.001; Figure 5d). All these results indicate that NPF regulates feeding behaviour, and the dsNPF transgenic cotton is a potential and efficient biotechnology for field control of *H. armigera*.

**Table 3:** Larval feeding inhibition on transgenic cotton plants expressing dsNPF

	dsGFP	dsNPF	*P* value	Decrease %
	*n* = 9	*n* = 9		
Area of leaf eaten (cm^2^)	18.280 ± 1.25	4.73 ± 0.99	<0.0001	74.13
Body length of larvae (cm)	2.27 ± 0.05	1.77 ± 0.05	<0.0001	21.87
Body weight of larvae (g)	0.34 ± 0.01	0.24 ± 0.02	0.003	29.76
Mortality rate after closion (%)	0	100	<0.0001	—

### Effects of dsNPF on regulating energy metabolism

**Figure 6:**
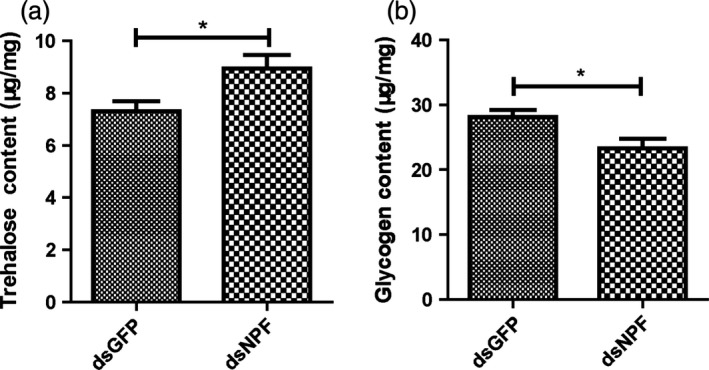
Larvae energy metabolism affected by applications of dsNPF. Glycogen and trehalose were tested at 72 h after injection of dsNPF RNAi (*n* = 30). (a) Trehalose content in dsNPF or dsGFP RNAi‐treated larvae (*P *< 0.05). (b) Glycogen content in dsNPF RNAi‐treated larvae compared with control counterparts (*P *< 0.05). Differences at “*” P < 0.05.

To understand how *npf* regulates feeding, we applied dsNPF to fifth‐instar larvae and measured their body trehalose, glycogen and total lipid. Results showed that dsNPF caused significant increases of trehalose and decreases of glycogen (Figure 6a,b). However, it had no effects on total lipid (Figure S4). These results suggest that *npf* regulates feeding behaviour to reduce the metabolism of glycogen to produce trehalose, potentially because feeding provides nutrients that reduce the need for metabolism of stored glycogen.

## Discussion

In this study, we found that *Harmnpf*1 and *Harmnpf*2 were encoded by the same gene, which has been recognized as an interesting aspect in gene regulation (Leff and Rosenfeld, 1986). The biological significance for alternative splicing is to produce a diversity of proteins (Black, 2003). The insect NPF was first purified in 1998 and completely sequenced in 2011 (Huang *et al*., 2011). The precursors of NPFs include a signal peptide, a propeptide and a C‐terminal peptide (Maule *et al*., 1995)—a result consistent with our findings.

We found that the expression levels of *H*. *armigera npf1* and *npf2* are different, because *npf2* levels were lower than those of *npf1* (Figure 1a). This case is similar to adipokinetic hormone (AKH) in locust (Vroemen *et al*., 1997). *npf* is highly expressed in midgut, and is also expressed in brain, suboesophageal ganglion, foregut and hindgut, as has been previously reported for *R. flavipes*,* H. zea* and *D. melanogaster* (Huang *et al*., 2011; Nuss *et al*., 2010; Veenstra and Sellami, 2008). In addition, *npf* is highly expressed at the larval 1st instar (just after emerging as larvae from egg shells) and the 5th instar (the larval gluttony stage), which suggests that the first‐instar larvae also are an important feeding period for their growth and development, in addition to the fifth‐instar gluttony stage. From previous reports, NPF has important roles in resisting low temperature and noxious food in *D. melanogaster* (Lingo *et al*., 2007; Wu *et al*., 2005).

The feeding regulation of NPF has been assessed in *D. melanogaster*, a Dipteran insect. However, the possible roles of NPFs in regulating feeding in Lepidoptera have not yet been reported, even though many of them are important economic and agricultural pests. In this study, we demonstrated that the *npf* of *H*. *armigera* is associated with increased feeding when *npf* is increased after food deprivation. On the contrary, knocking *npf* down by both applications of dsNPF (the transgenic tobaccos transiently expressing dsNPF and the transgenic cottons stably expressing dsNPF) had impacts on feeding inhibition, with reduced relative food consumption rate and growth rate, and smaller body size and weight. The damage to the transgenic cotton plants by cotton bollworms seems to be effectively reduced by 74%. Importantly, the NPF RNAi results in high mortality of this insect, in which all individuals did not normally pupate or emerge because of undeveloped bodies (Table 3). Therefore, *npf* is very critical for feeding regulation of *H*. *armigera*. This new type of biotechnological product could bring about a potential control of the cotton bollworm, *H*. *armigera*.

In *D. melanogaster*, a previous report showed that the NPF system is regulated by insulin, through the InR (insulin receptor)/PI3K/S6K pathway (Wu *et al*., 2005). However, how NPF regulates feeding is still unclear. In this study, we found that changed larval feeding behaviour by the dsNPF may cause changed metabolism, with a decrease of glycogen and an increase of trehalose. In other words, the decrease in feeding which results from NPF knockdown causes changes in glycogen and trehalose levels. Presumably with less feeding, more stored glycogen has to be used to break down to trehalose for energy supply of *H*. *armigera*.

## Experimental procedures

### Insects rearing

The *H*. *armigera* colony used in this study was established from insects collected in Zhengzhou, Henan Province, and has been maintained in the laboratory since 2007. They were reared at 25 °C and 65% relative humidity (RH) under a photoperiod of 16L : 8D with an artificial diet described by Wu and Gong (1997).

### Construction of cDNA library

Brains from fourth‐instar larvae were dissected in PBS treated with diethyl pyrocarbonate (DEPC), then frozen immediately in liquid nitrogen and stored at −80 °C until use. Total RNA was extracted with TRIzon reagent (TIANGEN, Beijing, China). For the specific methodology, see Liu *et al*. (2013).

### Full‐length cDNA cloning of *npf*

The full‐length *npf* cDNAs were obtained by the rapid amplification of cDNA ends (RACE) technique using the DNA from the cDNA libraries as PCR templates. For 5′ RACE, the degenerate reverse primers (5′ RACE1F) were designed according to the conserved region (NPF: QAARPRFGKR). Plasmid libraries (0.1 μL) were used as templates with *LA* Taq^®^ (Takara, Dalian, China) using forward primer 5′ SMART; reverse primer 5′ RACE1F. For the specific methodology, see Liu *et al*. (2013).

### Identifying open reading frames (ORF) of *HarmNPF*

The full‐length genes were amplified using the specific primers (FSP1 and NPF‐R), which were designed according to the 5′ and 3′ RACE sequences (Table S1). Conventional PCR was applied using 1 μL of cDNA (first Strand cDNA synthesized above). Reaction mixtures contained 1.25 U (5 U/L) *LA* Taq^®^, 2.5 μL 10× *LA* buffer solution and 4 μL dNTP (2.5 mm each) in a final volume of 25 μL. For the touchdown PCR conditions, please see Liu *et al*. (2013). The longest band for each gene was eluted from the gel using the E.Z.N.A^®^ Gel Extraction kit (OMEGA, Bio‐Tek, Inc., Norcross, GA, USA), and then directly cloned into the pGM‐T vector (TIANGEN, Beijing, China). The clone was sequenced twice in both directions using M13 forward and M13 reverse primers.

### Bioinformatic analysis

The *H. armigera npf* signal peptides were predicted using the online program SignalP 3.0: http://www.cbs.dtu.dk/services/SignalP/ (Bendtsen *et al*., 2004). Prohormone cleavage sites were predicted based on previously established protocol (Southey *et al*., 2008) using the website: http://neuroproteomics.scs.illinois.edu/cgi-bin/neuropred.py (Southey *et al*., 2006). For the specific methodology, see Liu *et al*. (2013).

### Spatiotemporal expressions of *Harmnpf*

To monitor transcriptional levels of *Harmnpf* from different larval tissues, we explored the semi‐quantitative reverse transcription–PCR (RT‐PCR). Total RNA was extracted from the brains (Br), suboesophageal ganglia (SG), thoracic ganglia (T), abdominal ganglia (A), foregut (FG), midgut (MG) and anterior hindgut (HG) as described. Tissue distribution of *Harmnpf* was investigated by RT‐PCR according to Chen *et al*. (2007) and Yang *et al*. (2010). For the specific methodology, see Liu *et al*. (2013).

To determine the expression of *Harmnpf* at different developmental stages of larvae, total RNA from larval whole bodies was prepared using TRIzol (TIANGEN), and cDNA was further synthesized using the RealMasterMix System for qRT‐PCR. The qRT‐PCR was used to quantify the levels of *npf* (*Harmnpf2* was selected for the qPCR throughout the manuscript) and actin as a control. The experiments were performed in triplicates. Data analysis was performed using ABI Stepone software (Applied Biosystem, Foster, CA).

### Relationship between food deprivation and *npf*

To further determine that *npf* impacts larval feeding, we designed a food deprivation experiment. After 24 h of normal feeding, the fifth‐instar larvae were reared with agarose (food deprivation) and normal food separately. The larvae midguts were collected for quantitative analysis of *npf* expression after food deprivation for 24 h. The qRT‐PCR method and analysis were the same as above.

### Synthesis of double‐strand RNA (dsRNA**)**

The dsNPF was produced by *in vitro* transcription using the T7 RiboMAX™ expression system (Promega, Madison, Wisconsin, USA). To produce DNA templates for the synthesis of both dsNPF1 and dsNPF2, a T7 RNA polymerase promoter was added to the 5′‐end of the DNA sequence using PCR with their specific primers for dsNPF‐F & dsNPF‐R in Table S1. PCR products were purified by TIANgel Midi Purification Kit (Cat. #DP209‐02). The PCR products were quantified and used as templates to prepare dsRNA using RiboMAX™ kit. To anneal, RNA reactions were incubated at 70 °C for 10 min, and then slowly cooled down to room temperature (~20 min). The annealed dsRNA was treated with A RQ1 RNase‐Free DNase and precipitated by adding 0.1 volume of 3M Sodium Acetate (pH 5.2) and 1 volume of isopropanol. The dsRNA sample was resuspended in nuclease‐free water and stored at −20 or −70 °C.

### Effects of *NPF* by applications of dsNPF RNAi

Ten micrograms of dsNPF RNAi or dsGFP RNAi was respectively injected into the lateral intersegmental membrane between the third and fourth abdominal segment of the selected fifth‐instar larvae, and the incision was sealed immediately with wax at the injection point. Each group was performed in triplicates with 15 individuals at each repeat (*n* = 45 larvae/group). Both controls and treatments were reared with a certain amount of artificial diets renewed every day. After 24/48/72 h, the treated and control larvae were observed separately, with measurements including larval weight, remainder of the artificial diet and faeces. As food is fresh, it is also necessary to set a blank experiment as a control, measuring the weight change of diet caused by the change of water content. Larval food consumption is calculated by the following formulae from Scriber and Slansky (1981).

$$I=w-\left(L+\frac{aW+bL}{2}\right)$$

*I* is the food ingested (food consumption).
*W* is the initial weight of the food in experimental group.
*L* is the final weight of the food in experimental group.
$a=\frac{\text{the initial weight of the food in control group}\;-\;\text{the final weight of the food in control group}}{\text{the initial weight of the food in control group}}.$
$b=\frac{\text{the initial weight of the food in control group}\;-\;\text{the final weight of the food in control group}}{\text{the final weight of the food in control group}}.$
$\text{Relative consumption rate (RCR)}\;=\;\frac{I}{\bar{B}\times T}.$
$\text{Relative growth rate (RGR)}\;=\;\frac{B}{\bar{B}\times T}.$
$\overset{¯}{B}$ = mean weight during the time period.
*B* is the assimilated food used for growth (biomass gained).
*T* is the duration of feeding period (days).

### Construction of plasmids

Plasmids were constructed using standard cloning techniques. dsRNAi constructs were prepared by adding appropriate restriction sites to the ends of the primers used to perform PCR amplification with DNA polymerase (TIANGEN) and primers (p‐NPF‐F and p‐NPF‐R) in Table S1. The PCRs began with 94 °C denaturation for 3 min, then 35 cycles of denaturation at 94 °C for 30 s, 56 °C annealing for 30 s, and 72 °C extension for 1 min. The PCR products and pGreen‐HY104 vector were digested separately with restriction enzymes HindIII and EcoRI. They then were further purified, ligated and transformed into DH5a. The newly constructed plasmid was named dsNPF‐pGreen‐HY104 plasmid. The control plasmid dsGFP‐pGreen‐HY104 was constructed with the same method above, using primers for dsGFP.

### Preparation of transgenic tobacco

Tobacco seeds were planted in sterilized culture medium and were transplanted to the aseptic nutritive bowl after 7 days. Then, they were cultured at 25 °C with 16‐h light and 20 °C with 8‐h dark. Four to five‐leaf‐stage tobacco plants were chosen for infiltration with agrobacterium (*Agrobacterium tumefaciens*) containing the constructed plasmids (dsNPF‐pGreen‐HY104 and dsGFP‐pGreen‐HY104). For the detailed methodology of transgenic tobacco, see Yang *et al*. (2000).

### Cotton Preparation of transgenic cotton and larval feeding

The cotton strain 2047B was grown in the field until flowering. After self‐pollination for 24 h, the dsNPF‐pGreen‐HY104 plasmid was injected into the ovary by hypodermic syringe. The injected bolls continuously grew to produce mature seeds. These seeds were grown until two leaves had appeared. Genomic DNA from one was analysed to identity the positive strains by PCR using the primers (NPF‐F and NPF‐R) in Table S1.

For larvae feeding on cotton plants in the field, 10 larvae were randomly released on the top of mature leaves to feed on each plant for 3 days. The treatments were performed in triplicates with three individual plants for each repeat (*n* = 9 plants/treatment). The cotton plants were mesh‐enclosed to prevent insects climbing to other plants. The treatment was performed in triplicates with three individual plants for each repeat (*n* = 9 plants/treatment).

For larvae feeding on cotton leaves in the laboratory, the leaf area was scanned with a scanner (HP Deskjet 1050), and the leaf was then placed in a Petri dish with moist filter paper and a fifth‐instar larva was allowed to feed on the leaf. After every 24 h, the eaten leaf area was calculated by ImageJ (leaf was changed every 24 h). The feeding leaf area was calculated as the fresh leaf area (*S*_0_) minus the eaten leaf area (*S*_1_). The treatment was performed in triplicates with three larval individuals for each repeat (*n* = 9 larvae/treatment).

### Northern blot analysis

For the northern blot hybridization, total RNA was extracted from transgenic plants (cotton and tobacco) leaves with TRIzol^®^ reagent. Forty micrograms of total RNA from each sample was heated at 65 °C for 15 min, cooled on ice and loaded on 1.3% w/v agarose gels, electrophoresed in denaturing buffer containing formaldehyde at 50 V for 2 h and visualized using UV. RNAs were blotted on to nylon membranes with 10 × SSC and cross‐linked to membranes by UV cross‐linking. A α^32^P‐UTP labelled full‐length NPF riboprobe was generated by *in vitro* transcription (Maxscript kit; Ambion, Austin, TX, USA). Probe was added and hybridized overnight at 65 °C. Membranes were washed in wash buffer (0.1 × SSC, 0.1% SDS) once, then washed 3 times with 100 mL of prewarmed wash buffer for 20 min, each in a hybridization oven at 68 °C, after which hybridization signals were detected by X‐ray film (Kodak, Rochester, NY, USA).

### Southern blot analysis

Total genomic DNA was isolated from 1‐week transgenic cotton leaves. Fifty micrograms of the genomic DNA was digested with *Hind III*, and the DNA fragments were separated by electrophoresis in a 1.0% w/v agarose gel in 1 × TAE buffer for 12 h at 25 V. The gel was sequentially subjected to denaturation buffer (1.5 m NaCl and 0.5 m NaOH for 30 min) and neutralization buffer (1.5 m NaCl and 1 m Tris base for 30 min). The DNA was transferred to a nylon membrane using 10 × SSC buffer and UV cross‐linked. *npf* coding regions labelled with α^32^P‐CTP were generated using a Rediprime Labelling Kite (Prime‐a‐Gene Labelling System; Promega). The probe was added to hybridization buffer (Rapid‐hyb buffer) and incubated overnight at 65 °C. The hybridization buffer was eluted and the membrane rinsed with wash buffer (0.5 × SSC, 0.2% SDS) once. Then, the membrane was washed 3 times with 100 mL of prewarmed wash buffer for 20 min each in a hybridization oven at 68 °C. The radioactivity signal on the membrane was detected by X‐ray film (Kodak).

### Determination of total lipid, glycogen, trehalose

The microseparation of glycogen, trehalose and total lipid used the method described by Van Handel (1965) with a slight modification by Zhou *et al*. (2004). Whole‐body homogenates of each individual were used to extract glycogen, trehalose and total lipid, respectively. Glycogen and trehalose were measured using the anthrone method with glycogen and trehalose as standards, respectively (Sigma Chemical, St.Louis, MO, USA). Total lipid was quantified by the vanillin assay. Each independent experiment was performed with triplicates with 10 individuals in total for each replicate.

### Statistical analysis

All data were statistically analysed by one‐way ANOVA using the Statistical Package for the Social Sciences (SPSS), version 11.5 for Windows. More than two group data were analysed with one‐way ANOVA followed by the Tukey–Kramer HSD Test as the *post hoc* test.

## Conflict of interest

The authors declare no conflict of interest.

## Supporting information


**Figure S1** The expressions of *Harmnpf1* and *Harmnpf2* in tissues of 5th instar larvae.
**Figure S2** Construction of the transgenic tobaccos.
**Figure S3** Northern blot and Southern blot analyses of transgenic cotton and tobacco.
**Figure S4** Total lipid metabolism not affected by applications of dsNPF.
**Table S1** Primers for PCR templates.
