# Supplementary material for: Development of a novel‐type transgenic cotton plant for control of cotton bollworm
Source: Plant Biotechnol J. 2016 Feb 3;14(8):1747–55. doi: 10.1111/pbi.12534 (PMC5067616; doi:10.1111/pbi.12534)
Supplement: Supplementary file 1 — Figure S1 The expressions of Harmnpf1 and Harmnpf2 in tissues of 5th instar larvae. Figure S2 Construction of the transgenic tobaccos. Figure S3 Northern blot and Southern blot analyses of transgenic cotton and tobacco. Figure S4 Total lipid metabolism not affected by applications of dsNPF. Table S1 Primers for PCR templates. [file PBI-14-1747-s001.docx]

**Supplementary Table**

**Supplementary table 1 Primers for PCR templates**

| Application of primers | sequence |
| --- | --- |
| 5' RACE1F | TTNCCRAANCKNGGNCKIGCIGCYTG |
| FSP1 | ATGCTGAACAAGAACATCG |
| NPF-R | AAGACAACGTGAGGAGTAGAG |
| dsNPF2-F | ATGCTGAACAAGAACATCG |
| dsNPF2-R | CTGTTGCACTCCTCATCTC CTTCTGG |
| actin-F | GTGTGATGGTCGGTATGGGTCAGAAG |
| actin-R | CAGATCTTCTCCATGTCCTCCCAGT |
| p-NPF2F | GCCCGGGAATTCATGCTGAACAAGAACATCG |
| p-NPF2R | CGGATCCAAGCTTTCATCTC CTTCTGGCGTAC |
| NPF2-F | GTTTCGCTGTCCGTTACTCC |
| NPF2-R | GGTCAATCCTGGGTCTAGCA |

**Supporting Figures**


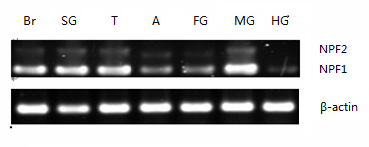


**Figure S1 The expressions of *Harmnpf1* and *Harmnpf2* in tissues of 5th instar larvae**

The RT-PCR profiles of *Harmnpf1* and *Harmnpf2* transcripts in different tissues of 5th instar larvae (Br-brain, SG-subesophageal ganglia, T-thoracic ganglia, A-abdominal ganglia, FG-foregut, MG-midgut, HG-anterior hindgut).

**
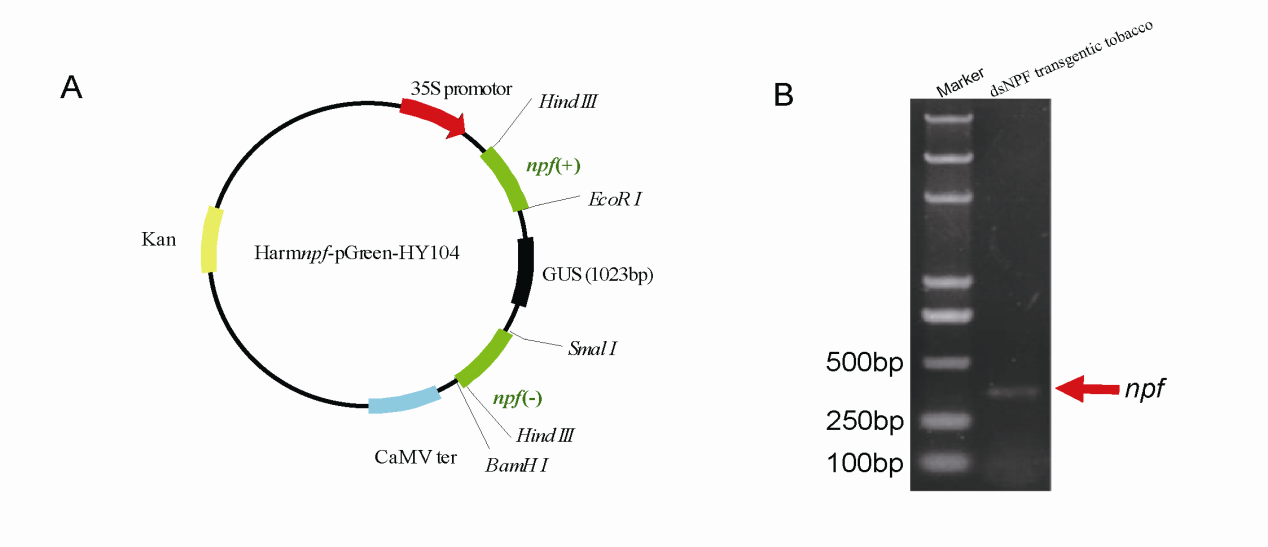
**

**Figure S2 Construction of the transgenic tobaccos**

**A** indicates the pGreen vector linked with dsNPF. **B** indicates the DNA identifications of dsNPF RNAi in transgenic tobaccos by PCR, in which Marker indicates standard molecular weight.


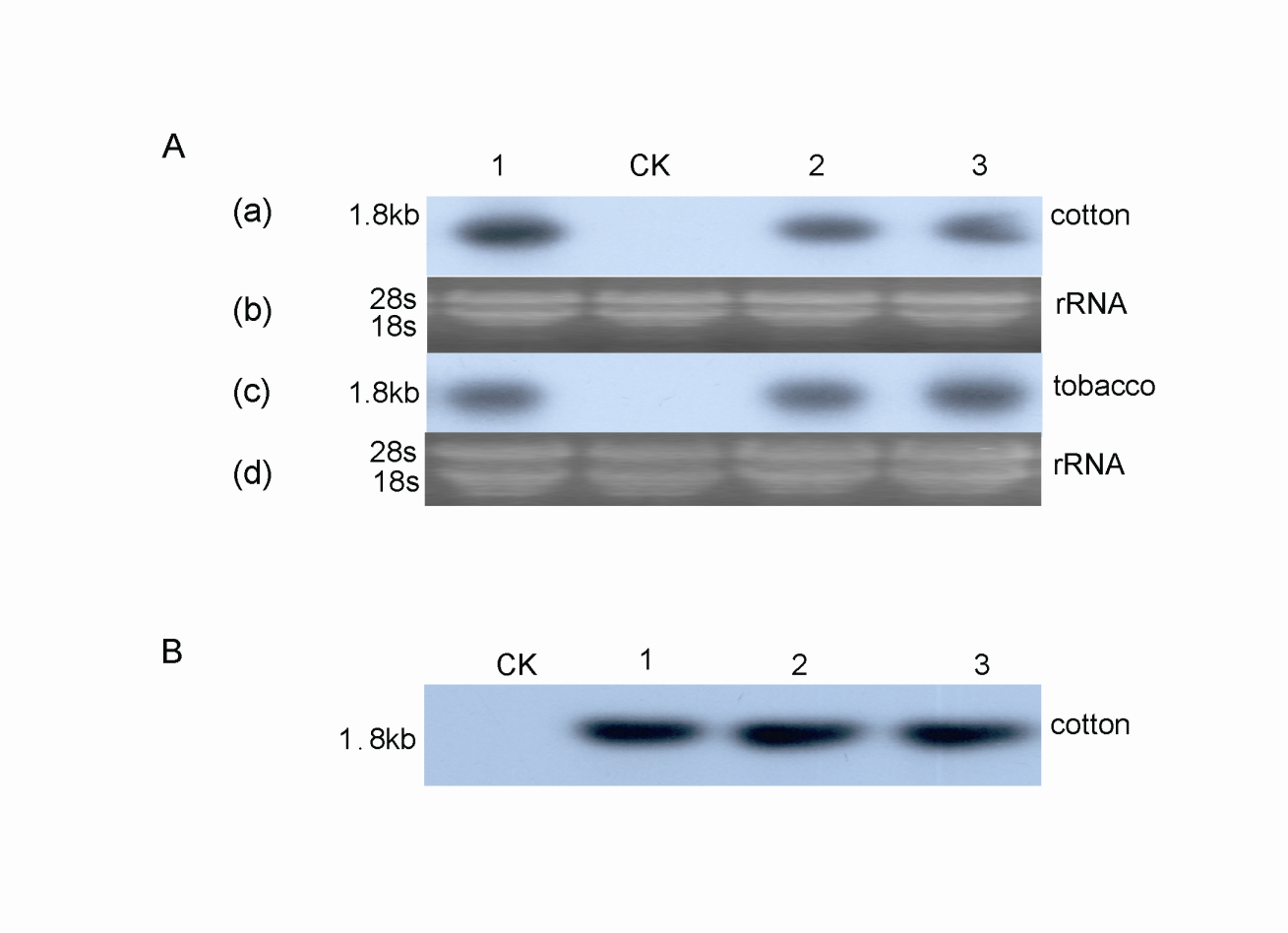


**Figure S3** **Northern blot and Southern blot analyses of transgenic cotton and tobacco**

Northern blot and Southern blot analyses of rRNA and genomic DNA from the leaves of T_0_ transgenic plants. 1, 2, 3 represent 3 events of dsNPF transgenic plants，CK was dsGFP transgenic plant. A. (a) and (c) represent Northern blot hybridization results for dsNPF transgenic cotton and tobacco. (b) and (d) represent ethidium bromide stained ribosomal RNA as a guide for loading control equivalence. B represents southern blot hybridization results for dsNPF transgenic cotton. Total genomic DNA was isolated from one week transgenic cotton leaves. Genomic DNA was digested with *HindIII* and probed with *npf* coding sequence.


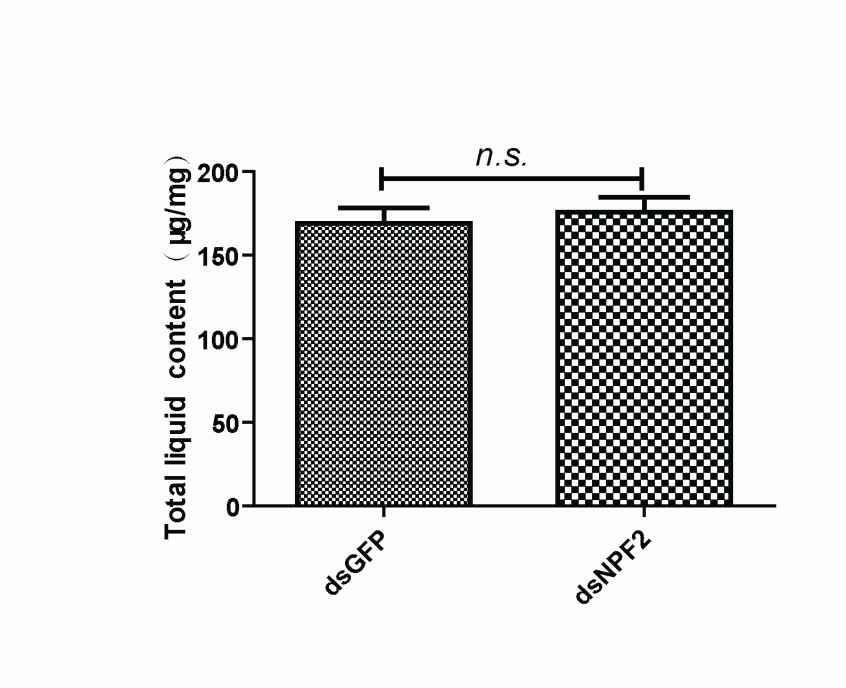


**Figure S4** **Total lipid metabolism not affected by applications of dsNPF**

Total lipid was tested at 72 hours after injection of dsNPF RNAi (n=30). The result indicated that there was no significant difference in total liquid content between dsNPF and dsGFP injected larvae.
